# Oral rehabilitation with dental implants in patients with recessive dystrophic epidermolysis bullosa: A retrospective study with 2-15 years of follow-up

**DOI:** 10.4317/medoral.23331

**Published:** 2020-01-22

**Authors:** David Peñarrocha-Oltra, Rubén Agustín-Panadero, Blanca Serra-Pastor, María Peñarrocha-Diago, Miguel Peñarrocha-Diago

**Affiliations:** 1 DDS, PhD. Adjunct Lecturer, Unit of Oral Surgery, Department of Stomatology, Faculty of Medicine and Dentistry, University of Valencia, Valencia, Spain; 2 DDS, PhD. Adjunct Lecturer, Unit of Prosthodontics and Occlusion, Department of Stomatology, Faculty of Medicine and Dentistry, University of Valencia, Valencia, Spain; 3 DDS. Associate Professor, Unit of Prosthodontics and Occlusion, Department of Stomatology, Faculty of Medicine and Dentistry, University of Valencia, Valencia, Spain; 4 MD, DDS, PhD. Full Professor, Unit of Oral Surgery, Department of Stomatology, Faculty of Medicine and Dentistry, University of Valencia, Valencia, Spain; 5 MD, DDS, PhD. Chairman of Oral Surgery, Department of Stomatology, Faculty of Medicine and Dentistry, University of Valencia, Valencia, Spain

## Abstract

**Background:**

Epidermolysis bullosa (EB) comprises a group of hereditary disorders characterized by mechanical fragility of the skin and mucous membranes, with the development of blisters and vesicles in response to minimum tissue friction. Recessive dystrophic epidermolysis bullosa (RDEB) with generalized involvement is the most common subtype in the oral cavity. The present study was carried out to investigate dental implant survival, peri-implant tissue condition, patient satisfaction, and the impact of treatment upon the quality of life of patients with RDEB rehabilitated with implants and full-arch implant-supported prostheses.

**Material and Methods:**

Thirteen patients with RDEB underwent dental implant treatment between September 2005 and December 2016. A retrospective study was made to analyze implant survival, peri-implant tissue health and patient satisfaction.

**Results:**

A total of 80 implants were placed (42 in the maxilla and 38 in the mandible) in 13 patients between 20-52 years of age and diagnosed with RDEB. All the implants were rehabilitated on a deferred basis with 20 full-arch prostheses. Fifteen fixed prostheses and 5 implant-supported overdentures were placed. The implant survival rate was 97.5% after a mean follow-up of 7.5 years after prosthetic loading. Fifty percent of the implants showed mucositis at the time of evaluation. Probing depth was maintained at 1-3 mm in 96.2% of the implants, and bleeding upon probing was observed in 67.5% of the implants. There was a high prevalence of bacterial plaque (85%).

**Conclusions:**

The treatment of edentulous patients with RDEB by means of implants and implant-supported prostheses is predictable as evidenced by the high success rate, and improves patient self-esteem and quality of life.

** Key words:**Epidermolysis bullosa, dental implants, implant-supported prostheses.

## Introduction

Epidermolysis bullosa (EB) comprises a group of inherited disorders characterized by mechanical fragility of the skin and mucous membranes, with the recurrent development of blisters and vesicles in response to the slightest friction (1).

The latest congress on the diagnosis and classification of the disease (Vienna 2007) classified EB into four groups and 32 subtypes. Among these groups, recessive dystrophic epidermolysis bullosa (RDEB) with systemic involvement is the subtype most commonly found in the oral cavity. Recessive dystrophic epidermolysis bullosa manifests as blisters all over the body, especially in areas of friction such as the hands, feet, elbows and knees (1,2). Such blisters usually lead to very painful ulcers that heal producing scars which in turn cause soft tissue contraction. Patients usually present syndactyly due to the continuous friction suffered by the hands in daily life, and also esophageal stenosis, which causes obstruction of the digestive tract and secondary dysphagia (1-3).

**Figure 1:**
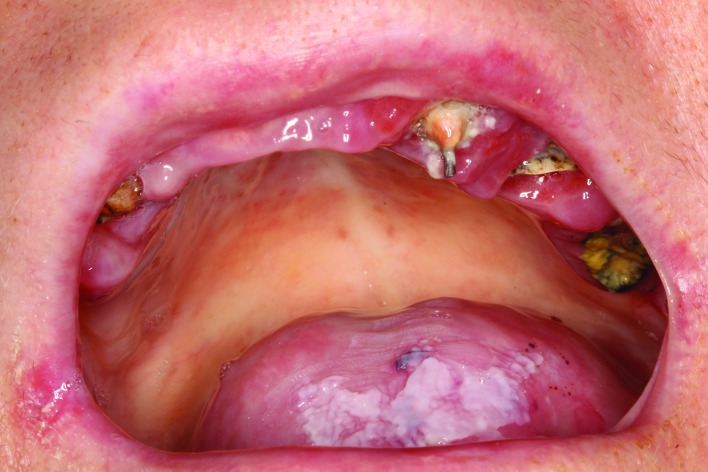
Pre-treatment intraoral view showing the presence of teeth destroyed by rampant caries. Lesions are seen at the corners of the mouth and on the buccal and lingual mucosa.

Regarding the oral manifestations of RDEB, the numerous blisters and ulcers result in scars that greatly alter the oral architecture, creating severe microstomia (limited mouth opening), ankyloglossia (adherence of the tongue to the floor of the mouth) and disappearance of the vestibule (2-4). Routine dental care proves very difficult in these patients, since any conventional brushing technique involves friction of the oral mucosa and thus causes the appearance of blisters and ulcers (Fig. 1).

For this reason, the presence of numerous and extensive caries and severe periodontal disease are very frequent in such patients, resulting in a loss of teeth at an early age (2).

The oral rehabilitation of these patients with dental implants has been shown to offer high success rates over the short term, with significant improvement in quality of life (5-19). A functional dentition allows patients to improve their nutritional status (11) and reduces the potential for oral and esophageal soft tissue damage thanks to more efficient mastication (12,13). Moreover, dental implants can restore esthetics and self-esteem to these generally young patients. However, there are no data in the literature on the long-term success of dental implants or on the maintenance of peri-implant tissue health over time in patients with RDEB.

The aim of the present study was to evaluate implant and prosthetic treatment outcomes, peri-implant tissue health status and the satisfaction and quality of life of patients with RDEB rehabilitated using dental implants after a minimum follow-up of two years.

## Material and Methods

A retrospective single cohort study was carried out in patients with RDEB treated with dental implants at the Oral Surgery and Implantology Clinic of the University of Valencia (Valencia, Spain) up until June 2018. Part of the patient sample included in the present study has been reported after shorter follow-up periods in several previous publications (7,9,10,14-16).

The selection criteria were: 1) patients diagnosed with RDEB; 2) edentulous or partially edentulous individuals (with teeth requiring extraction due to severe caries or periodontal disease); 3) treatment with dental implants and full-arch fixed prosthesis or overdentures; and 4) a minimum follow-up period of two years after prosthetic loading.

The study was approved by the Ethics Committee of the University of Valencia with a procedure number H1404212828195. All the patients gave written informed consent before the treatment, with specification that their data could be used for research purposes.

Before surgery, radiographic preoperative evaluation had been performed using panoramic radiographs and cone beam computed tomography (CBCT) scans. Prior to surgery, the lips of the patient were lubricated with vaseline, in the same way as the surgical instruments, to avoid damaging the mucosa.

All surgeries had been carried out under conscious intravenous sedation using 1% propofol (Diprivan, Astra-Zeneca Pharma, Madrid, Spain) administered by an anesthetist. Local anesthesia was performed using 4% articaine and adrenaline 1:100,000 (Ultracain®, Aventis Pharma, Bad Soden, Germany), infiltrating the anesthetic solution deep into the tissues. Maxillary implants were placed combining drills and osteotomes to preserve the remaining bone and facilitate primary stability. In the mandible, conventional drilling was performed using the minimum necessary irrigation to reduce the use of aspiration. Three different implants were used: Phibo TSA® (Phibo Dental Solutions, Sentmenat, Barcelona, ​​Spain), Straumann Tissue Level® with SLA Surface (Straumann Institute, Waldenburg, Switzerland) and Ticare Inhex® implants (Mozo Grau, Valladolid, Spain).

Peri-implant defects were regenerated with particulate autologous bone collected during drilling, combined with tricalcium beta-phosphate (Kera-Os®, Keramat, Coruña, Spain), when the quantity of autologous bone proved insufficient. Resorbable collagen (Lyostypt®, B. Braun, Aesculap, Tuttlingen, Germany) was used to protect the particulate bone grafts.

Panoramic radiographs were taken immediately after surgery, and oral antibiotics (Clamoxyl®, Glaxo-Smith-Kline; 500 mg every 8 hours for 7 days) and nonsteroidal antiinflammatory drugs (Ibuprofen, Bexistar, Bacino Laboratory; 600 mg every 8 hours for 3 days) were prescribed.

**Figure 2:**
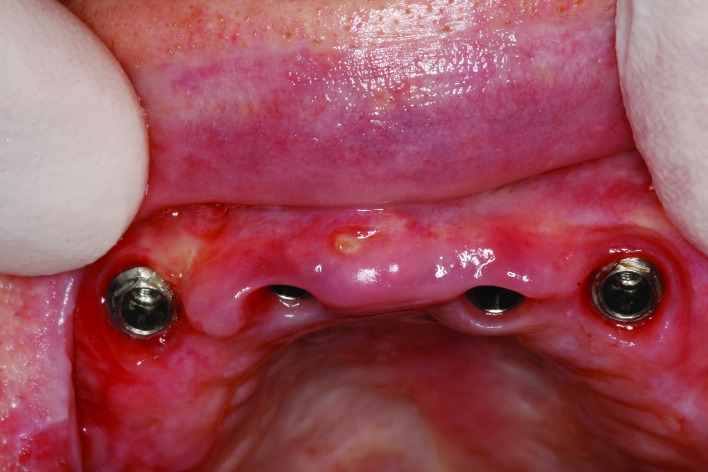
Keratinized periimplant mucosa after osseointegration of implants placed in the premaxillary zone.

The implants remained until three months after placement in the mandible and 6 months in the maxilla (Fig. 2).

**Figure 3:**
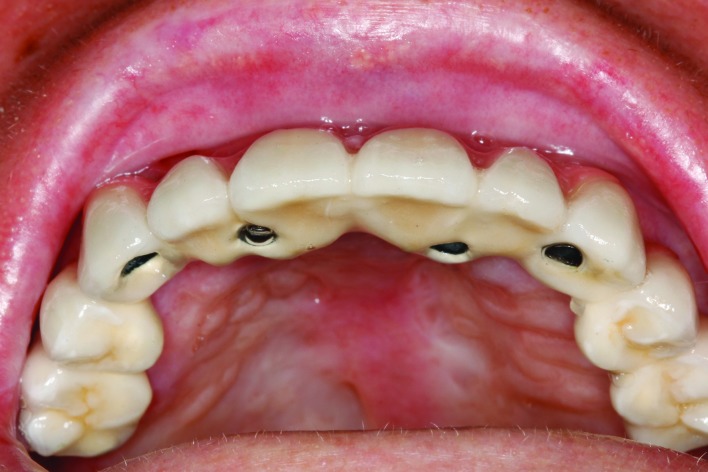
Post-treatment intraoral view of the fixed upper full-arch prosthesis; designed with dynamic abutments and fixation screws that can be tightened from the buccal side at an angle of 25-30º.

Prosthetic rehabilitation was performed using screw-retained or cemented implant-supported fixed prostheses or overdentures (Fig. 3).

When possible, clinical monitoring was carried out one and three months after surgery, and every 6 months after prosthetic loading. Panoramic radiographs were obtained every 12 months to assess peri-implant marginal bone loss. Intraoral periapical radiographs could not be performed, as they would damage the soft tissues. Bone loss was controlled with the Rhinoceros® software (Robert McNeel & Associates. Seattle, USA); comparing the initial radiography (radiography of the day of implant placement) with revision radiography. Following the ideal monitoring and maintenance program was not possible in all cases, since many of the patients had important physical limitations or lived in other cities or countries.

During the last control visit, a predefined protocol was used to register implant and prosthetic treatment failure, peri-implant tissue health status and patient satisfaction. Implants with mobility were removed due to progressive marginal bone loss or infection, and non-usable implants due to mechanical complications were considered implant failures. Prosthetic treatment was considered a failure if it caused pain or discomfort or was not useful for restoring patient oral function (mastication, phonation, esthetics).

Peri-implant tissue health status was assessed based on the following variables: 1) inflammation (redness and swelling); 2) the presence of suppuration; 3) the presence of plaque; 4) maximum probing pocket depth (PPD) measured with a millimetered periodontal probe (PCPUNC156, Hu-Friedy, Des Plaines, IL, USA) and classified into > or 3 mm; 5) bleeding on probing (BoP); 6) gingival recession, measured with a millimetered periodontal probe (PCPUNC156); and 7) the presence of keratinized mucosa (KM). Peri-implant marginal bone loss (< 2 mm or 2 mm) was assessed from panoramic radiographs, as intraoral radiographs were not available. With this information, the peri-implant tissue health status of each implant was classified as healthy, mucositis or peri-implantitis.

Patient satisfaction was evaluated using visual analog scales (VAS) from 0-10 with respect to the following aspects: general satisfaction, eating, speaking, esthetics, cleansibility, hygiene, comfort and self-esteem. The quality of life was assessed with a questionnaire that we made exclusively for this type of patients; using the OHIP questionnaire on quality of life as a reference. We thought that a special questionnaire was necessary due to the characteristics of EB patients; asking questions which the patient will compare his current situation (after treatment) with the initial one. Each question was answered with the following options: never, rarely, occasionally, very frequently.

## Results

Thirteen patients (9 women and 4 men) that had received 80 implants (42 in the maxilla and 38 in the mandible) were included in the study. The age of the patients at last follow-up ranged between 20-52 years (mean 33). Seven patients were treated of both arches. Fifteen implant-supported fixed prostheses, of which 5 were screwed and 10 cemented, and 5 overdentures were used. The mean follow-up period was 7.7 years (range 2-15 years) after prosthetic loading.

Two implants failed (2.5%) - both located in the mandible. One failed during the osseointegration period and the other was removed due to mobility detected 12 months after prosthetic loading. The clinical survival rate of the implants was 97.5%. With regard to the peri-implant tissues, signs of inflammation were observed in 50% of the implants at the time of the clinical evaluation. In 85% of the cases bacterial plaque was identified on the prosthesis and the implant prosthetic platform. Bleeding upon probing was noted in 67.5% of the implants. In turn, peri-implant probing depth was maintained at 1-3 mm in 96.2% of the implants, though 52.5% of the implants showed 0 mm retraction of the peri-implant mucosa. The presence of keratinized mucosa in the buccal zone of the osseointegrated implants was noted in 62% of the cases; 40% with a width of the keratinized mucosa of 2-3 mm; and a 22% with a width of was 1 mm. The remaining 38% showed no keratinized mucosa (mobile peri-implant soft tissue) (Table 1, Table 2).

Peri-implant bone loss after 7.7 years of follow-up was 1.65 0.54 mm as determined from the panoramic radiographs.

With regard to patient satisfaction after treatment, the mean score was over 9 for all the parameters evaluated (mastication, phonation, esthetics, comfort and self-esteem) except hygiene, which yielded a score of 6-8. The quality of life questionnaire after fitting of the prosthesis also yielded positive results. Specifically, none of the patients claimed to feel ashamed of their mouth after treatment, and 76.9% rarely or never felt unable to go about their daily life routines normally (Table 3).

**Table 1:**
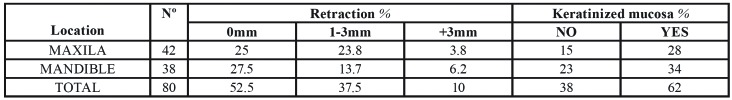
Peri-implant soft tissue health: Quantity of retraction and keratinized mucosa.

**Table 2:**
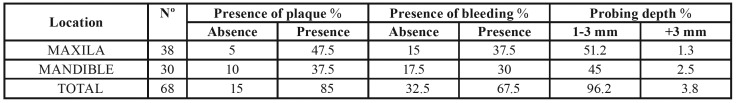
Peri-implant tissue health: Presence of plaque, presence of bleeding and probing depth.

**Table 3:**
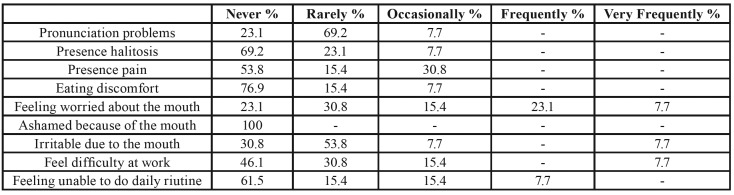
Quality of life.

## Discussion

Implant-supported prosthetic rehabilitation of edentulous patients with recessive dystrophic epidermolysis bullosa (RDEB) has been shown to afford high success rates (2,3,5) and is well tolerated by the patients. However, few data are available on the evolution of the peri-implant tissues over time.

According to the literature, the success rate of dental implants placed in patients with RDEB ranges from 97-100% (3,4,7). In our series we decided to assess implant survival in the mouth (97.5%) instead of the success rate, since the purpose of placing implants in patients of this kind is to afford function capable of ensuring improved nutrition. The literature makes no mention of the peri-implant tissues in patients with RDEB. In our study we assessed the peri-implant tissues based on measurements of gingival retraction, the presence or absence of keratinized mucosa and of bacterial plaque, and bleeding on probing and probing pocket depth. The results proved positive considering the skin and mucosal membranes of these patients, characterized by blisters that evolve towards ulcerations and retractile scars with substance loss. It is therefore clear that the peri-implant tissues suffer retraction associated to the prosthesis-implant gap, due to the ease with which bacteria accumulate at this interface. Mention also must be made of the problems these patients have for maintaining adequate oral hygiene. Many of them have no hands or fingers, and others suffer digital atrophy with syndactyly. Microstomia and ankyloglossia are moreover also observed. All this complicates the mechanical removal of bacterial plaque and explains the high prevalence of plaque end bleeding observed (85% and 67.5%, respectively) (2,3,4). Literature recommends the use of small and soft brushes, together with arms adapted for patients with physical limitations, or the prescription of chlorhexidine rinses as coadjuvant treatment (4).

The peri-implant bone loss observed (1.65 0.54 mm) was greater than the values considered normal in patients without systemic disorders (0.06 ± 1.11 mm) (18). Nevertheless, the use of implants was justified, since their clinical survival rate was 97.5%. Oral rehabilitation with implants in these patients results in improved mastication and swallowing, thereby stimulating the function of the stomatognathic system and optimizing food digestion. This in turn contributes to avoid esophageal stenosis secondary to the non-ingestion of solid foods. Furthermore, general patient health is improved, since being able to chew and swallow varied types of foods helps improve nutritional status (19).

With regard to patient satisfaction and quality of life, a study carried out by Peñarrocha-Oltra (7) on implant-supported fixed prostheses in patients with RDEB recorded a mean score of 9 for all the variables evaluated (comfort, function, esthetics, phonation and self-esteem). These results are consistent with those found in the literature (3,4). In our series we obtained a high score (9-10 points) for most parameters except oral hygiene, since the physical limitations inherent to RDEB, and the mucosal sequelae of brushing, greatly complicate correct hygiene.

The prosthodontic management of these patients has experienced changes in recent years. Because of the microstomia characterizing RDEB and the very high risk of aspiration of prosthetic elements and drivers, the prosthesis used were cemented on premanufactured titanium abutments. The limited oral opening, added to the difficulty of obtaining a good impression, made it necessary to prepare a prosthesis with a more permissive fit for passive adjustment of the structure, and which did not pose oral opening problems when manipulated in the mouth. Cemented prostheses require less oral opening for manipulation, since we do not need to insert the driver upon fitting the prosthetic structure.

The prosthesis can be fitted in sequence (first screwing the abutments and then cementing the superstructure - the driver only being needed when placing the abutments). The main problem with prostheses of this kind is the difficulty of removing them. These patients are unable to observe correct oral hygiene, and the cemented prosthesis does not facilitate periodontal maintenance.

Agustín-Panadero (19) have described a new prosthetic alternative for these patients in the form of prostheses directly screwed to the implant platform produced by CAD/CAM processing with dynamic abutments allowing the use of prosthetic fixation screws that can be tightened at an angle of 25-30 degrees. This means that the prosthetic structure can be screwed from outside the mouth, with no aspiration risk and no need to force oral opening. The screwed prosthesis is less voluminous than a cemented prosthesis and can be manipulated from outside the mouth. Furthermore, it can be removed each time the patient visits for periodontal maintenance, thereby improving the health of the peri-implant tissues. Since this design is very recent, only two patients in our study received this particular treatment.

The limitations of our study are the small sample size (13 patients) and the lack of uniform compliance with the patient follow-up visits and maintenance procedures. This is explained by the fact that many of the patients lived in other cities or countries, and the physical limitations (wheelchair or treatments in the form of dialysis) prevented many of them from reporting to all the scheduled visits.

## Conclusions

The results obtained in this study suggest that the treatment of edentulous patients with RDEB by means of implants and implant-supported prostheses is predicTable as evidenced by the high success rate, and improves patient stomatognathic function, self-esteem and quality of life.
